# Multifocal ERG Guiding Therapy in a Case of Hydroxychloroquine Premaculopathy

**DOI:** 10.1155/2015/656928

**Published:** 2015-10-18

**Authors:** José Antonio Sáez-Moreno, Concepción Domínguez-Hidalgo, José Manuel Rodríguez-Ferrer

**Affiliations:** ^1^Laboratory of Visual and Cognitive Neuroscience, Institute of Neuroscience, Granada University, 18100 Granada, Spain; ^2^Department of Clinical Electrophysiology, San Cecilio University Hospital, 18100 Granada, Spain; ^3^Department of Ophthalmology, San Cecilio University Hospital, 18100 Granada, Spain; ^4^Department of Physiology, School of Medicine, Granada University, 18100 Granada, Spain

## Abstract

We report the case of a 28-year-old female treated for systemic lupus erythematosus with hydroxychloroquine (200 mg/day) for 11 years. She was visually asymptomatic, with normal fundus appearance, normal colour vision testing findings, 20/20 visual acuity in both eyes, and only mild central bilateral defects on 10-2 perimetry. Multifocal electroretinography (mfERG) showed low density values for ring 1 in both eyes. Because the patient had not previously responded to alternative treatments and in consultation with her physician, the hydroxychloroquine dose was reduced to 200 mg four days/week. Four serial mfERGs performed at 4, 18, 25, and 34 months after dose reduction showed a progressive improvement in the definition and density of the responses until they were normalized at the third mfERG (25 months after hydroxychloroquine dose reduction). The fourth and final mfERG at 34 months confirmed the recovery in both eyes. Perimetry defects were mostly normalized. These results demonstrate the importance of mfERG for the safe management of patients under long-term hydroxychloroquine treatment.

## 1. Introduction

Hydroxychloroquine is commonly prescribed for the treatment of rheumatic diseases, particularly systemic lupus erythematosus and rheumatoid arthritis. However, hydroxychloroquine can cause toxicity in the outer retina and the retinal pigment epithelium, producing visual loss [1]. In some cases, these toxic effects continue after withdrawal of the drug [2]. The risk of hydroxychloroquine retinopathy is less than 0.5% but markedly increases to around 1% after 5–7 years of treatment and may be higher after 15–20 years of exposure to the drug [3, 4]. Previous studies showed that hydroxychloroquine produces a gradual decline in the density of multifocal electroretinography (mfERG) responses and that these alterations may improve after the cessation of treatment [5, 6]. The early detection of hydroxychloroquine-induced mfERG abnormalities can help to prevent the irreversible lesions that characterize the retinal toxicity of this drug [7]. We report a case in which a reduction in the hydroxychloroquine dose produced a nearly complete normalization of retinal function, as evidenced by serial mfERG assessment over a 34-month period in a patient treated with this drug for 11 years.

## 2. Case Report

**Figure 1:**
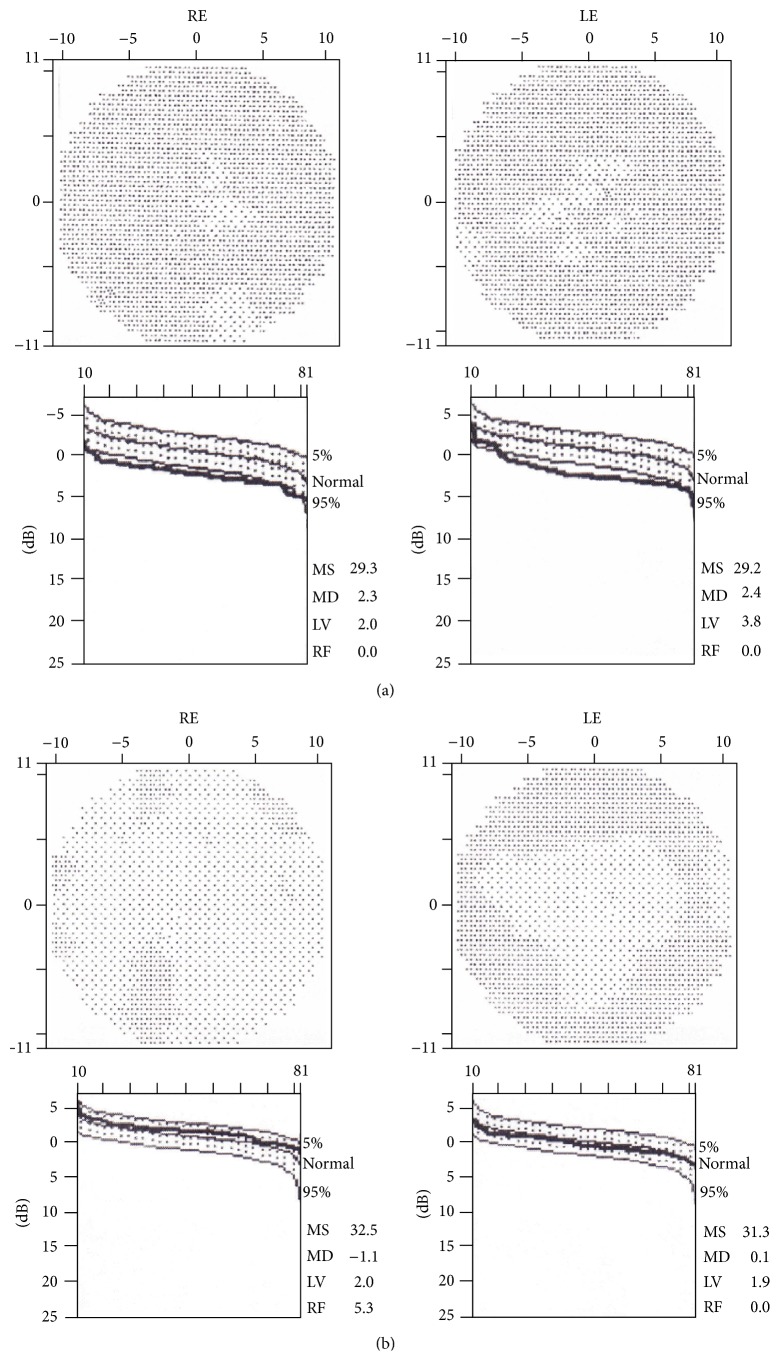
Perimetry 10-2 octopus and retinal sensitivity curve (below) performed before (a) and 34 months after (b) hydroxychloroquine dose deduction. LE: left eye. RE: right eye.

**Figure 2:**
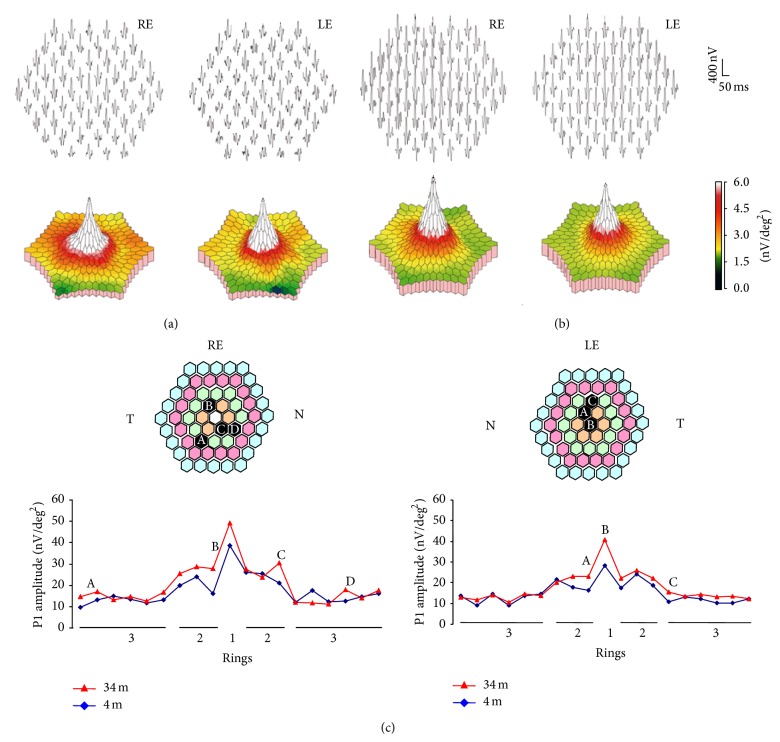
Trace array of 61 individual mfERG responses and three-dimensional topography response density plot (below) performed at 4 months and (b) 34 months after hydroxychloroquine dose reduction. The follow-up with mfERG showed a generalized increase in response amplitudes, particularly of the central ring. (c) P1 amplitudes of the responses obtained in rings 1–3 in the first (shown in (a), red line) and last (shown in (b), blue line) mfERG performed. Hexagons with increments higher than 40% in the follow-up are marked (A–D). LE: left eye; m: months; N: nasal; RE: right eye; T: temporal.

**Figure 3:**
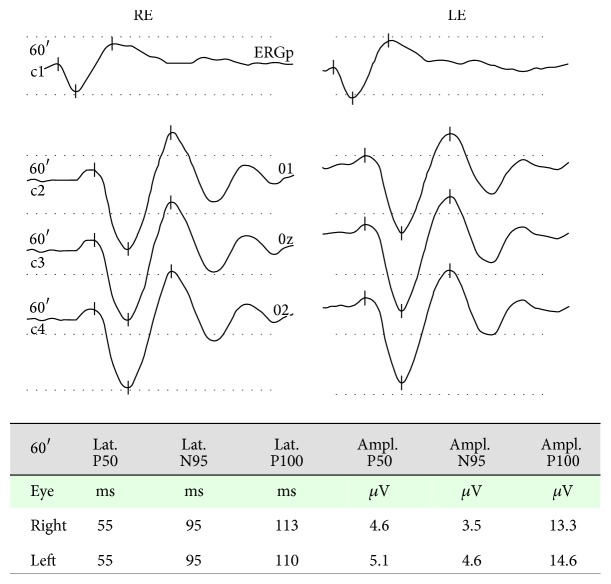
Pattern ERG-VEP recording of the patient, showing normal values of latencies and amplitudes of the different components. LE: left eye. RE: right eye.

In her annual control ophthalmic exam, a 28-year-old female (50 kg of body weight) treated for systemic lupus erythematosus with hydroxychloroquine for 11 years (4 mg/kg/day; 803 g of lifetime cumulative dose) presented for the first time with a mild sensitivity loss on 10-2 perimetry in both eyes (Figure 1(a)). The fundus appearance, colour vision testing findings, spectral domain optical coherence tomography (SD-OCT) scan, and fundus autofluorescence were normal. The patient had no visual symptoms, with 20/20 visual acuity in both eyes. Her medical history included autoimmune hepatitis and Raynaud's syndrome that had only responded to hydroxychloroquine. As a preventive measure, it was decided to temporarily reduce her hydroxychloroquine dose to 200 mg four days/week. The mfERG performed 4 months after the dose reduction showed low density values for ring 1 in both eyes, particularly in the left eye (Table 1, Figure 2(a)). The pattern ERG/VEP was found to be normal at 12 months after the dose reduction (Figure 3). The second mfERG, performed at 14 months after the first one, showed improvements in both eyes, supporting continuation of the treatment. Normal values were recorded 7 months later and were confirmed by the last mfERG at 34 months after dose reduction (Table 1, Figure 2(b)). Perimetry results showed similar improvements (Figures 1(b) and 2(c)). Multifocal ERG and pattern ERG were performed using a Diagnosys LLC system (Lowell MA, USA) according to International Society for Clinical Electrophysiology of Vision guidelines [4]. For mfERG testing the pupils were dilated fully. Refractive errors were corrected and a topical anesthetic was used to reduce discomfort and blinks. DTL electrodes were used for recording. The electrode impedance was less than 5 KΩ. Electrodes were placed always in the same position, just below the inferior border of the pupil and through both palpebral commissures, far from the inferior eyelid. Electrode position and fixation were monitored with a video camera and in case of poor fixation or electrode displacement last interval recording was erased and replaced for a new acquisition. Blink artifacts were automatically rejected. All the recordings used 61 pixels, spanning approximately 30° on either side of the fixation. Response densities of the first-order kernel of the mfERG in each patient were analyzed by grouping the 61 responses into five concentric rings. The amplitude of each ring response was measured between the first negative trough (N1) and the first positive peak (P1), yielding the N1-P1 response density in nV/deg^2^. Statistical analysis was performed using the SPSS 15.0.

## 3. Discussion

**Figure 4:**
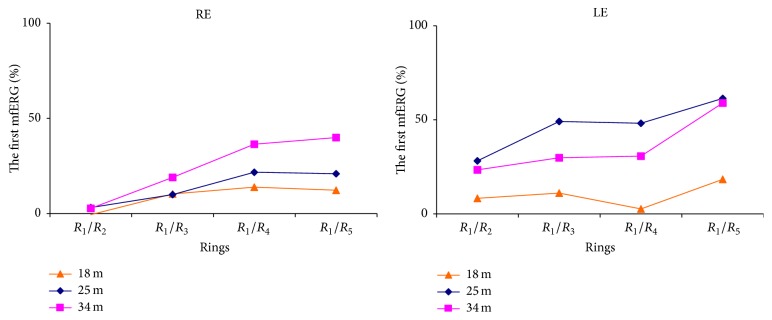
Percentage of change in rings ratios of mfERGs made at 18, 25, and 34 months when compared with those of the first mfERG, obtained 4 months after hydroxychloroquine dose reduction. LE: left eye. RE: right eye.

Hydroxychloroquine binds to melanin in the retinal pigment epithelium and blocks the attachment of autophagosome to lysosome, leading to a marked intracellular accumulation that is associated with retinal toxicity [9]. Hydroxychloroquine toxicity is clinically characterized by bilateral bull eye maculopathy, a ring of retinal pigment epithelium depigmentation that spares a foveal island. Although visual acuity may be excellent in these patients, most complain of difficulties in reading, decreased vision, missing central vision, glare, blurring, light flashes, and metamorphopsia [1]. Risk factors for retinal toxicity secondary to hydroxychloroquine include daily dosage >400 mg/day, cumulative dose >1000 g, treatment for >5 years, the presence of kidney or liver dysfunction or retinal disease, and aging [4]. The duration of treatment (11 yr) was a risk factor in the present patient, who had mild perimetric defects and bilateral low density of ring 1 in the mfERG. Because of the absence of symptoms and good visual acuity and the normal results of complementary examinations, she was diagnosed with hydroxychloroquine premaculopathy [10]. Given her previous failure to respond to treatments other than hydroxychloroquine, a decision was taken, in consultation with her internal medicine physician, to reduce the dose to 200 mg four days/week and to monitor her retinal function with mfERG. Studies in rhesus monkeys have shown that the earliest reversible histopathological changes occur in ganglion cells and in photoreceptor outer segments [11]. Although hydroxychloroquine may initially destroy ganglion cells, this was not the case in our patient, whose pattern ERG/VEP was normal. Hydroxychloroquine has been reported to produce a gradual attenuation in the density of mfERG responses that may improve after cessation of the treatment [5, 6]. Partial recovery after hydroxychloroquine withdrawal has also been demonstrated by SD-OCT [12]. Consistent with these reports, the present patient evidenced a progressive improvement in the density of mfERG responses in both eyes (Table 1), which reached normal values at 25 months after the hydroxychloroquine dose reduction. Normalization of the density and definition of N1-P1 complexes, which characterize mfERG responses, were confirmed in the final mfERG at 34 months after the dose reduction (Figures 2(b) and 2(c)). Rings ratios analysis (Table 2) shows also increments of the ratios throughout time (Figure 4), mainly in R1/R4-5, in accordance with the significant density increments in central rings (Table 1). The electrophysiological improvement of the 10-degree macular area was confirmed by independent perimetry (Figure 1). In their evaluation of the impact of the revised American Academy of Ophthalmology guidelines on screening for hydroxychloroquine retinopathy, Browning and colleagues suggested that a safe daily dose of hydroxychloroquine can be obtained through the longitudinal study of patients with serial mfERG assessments [13, 14]. This proposal is supported by the present case, in which serial mfERGs evidenced significant retinal recovery after reduction of the hydroxychloroquine daily dose, confirming the usefulness of long-term follow-up with mfERG to prevent toxicity by prescribing a safer dosage of hydroxychloroquine. However, it is important to be aware that conventional mfERG may fail to identify hydroxychloroquine toxicity in up to 28% of cases [15], while the specificity of mfERG to detect this toxicity has been estimated at 87% [14]. The present finding is unlikely to be a false positive, given that selective central perimetric and mfERG alterations were ameliorated by a reduction in the daily hydroxychloroquine dose, while the clinical conditions of the patient were preserved. Pharmacological control of systemic lupus erythematosus by hydroxychloroquine is of critical importance, because the retina may also be damaged by an inadequate dose of this drug.

## 4. Conclusions

This case demonstrates that (i) mfERG has sufficient sensitivity to detect hydroxychloroquine premaculopathy, (ii) retinal alterations of hydroxychloroquine premaculopathy can be reversed not only by drug cessation but also by dose reduction, and (iii) mfERG is useful to guide long-term hydroxychloroquine therapy.

## Figures and Tables

**Table 1:** P1 amplitudes (nv/deg^2^) follow-up.

	4 months		18 months		25 months		34 months	
	RE	LE	RE	LE	RE	LE	RE	LE
Ring 1	38.7^a^	28.2^a^	41.4	32.1^a^	41.9	43.8	49.1	40.9
Ring 2	22.1	19.3	23.8	20.3	23.2	23.4^*∗*^	27.3^*∗*^	22.7^*∗*^
Ring 3	13.5	11.9	13.1	12.2	13.3	12.4	14.4	13.3^*∗*^
Ring 4	10.0	8.2	9.4	9.1	8.9	8.6	9.3	9.1
Ring 5	8.6	8.0	8.2	7.7	7.7	7.7	7.8	7.3

^a^Values below normal [8]; ^*∗*^ *P* < 0.05  *versus* 4 months, Student's *t*-test; LE: left eye. RE: right eye.

**Table 2:** mfERG rings ratios analysis.

	4 months		18 months		25 months		34 months	
	RE	LE	RE	LE	RE	LE	RE	LE
*R*_1_/*R*_2_	1.8	1.5	1.7	1.6	1.8	1.9	1.8	1.8
*R*_1_/*R*_3_	2.9	2.4	3.2	2.6	3.2	3.5	3.4	3.1
*R*_1_/*R*_4_	3.9	3.4	4.4	3.5	4.7	5.1	5.3	4.5
*R*_1_/*R*_5_	4.5	3.5	5.0	4.2	5.4	5.7	6.3	5.6

## References

[1] Yam J. C. S., Kwok A. K. H. (2006). Ocular toxicity of hydroxychloroquine. *Hong Kong Medical Journal*.

[2] Mititelu M., Wong B. J., Brenner M., Bryar P. J., Jampol L. M., Fawzi A. A. (2013). Progression of hydroxychloroquine toxic effects after drug therapy cessation: new evidence from multimodal imaging. *JAMA Ophthalmology*.

[3] Mavrikakis I., Sfikakis P. P., Mavrikakis E. (2003). The incidence of irreversible retinal toxicity in patients treated with hydroxychloroquine: a reappraisal. *Ophthalmology*.

[4] Marmor M. F., Kellner U., Lai T. Y. Y., Lyons J. S., Mieler W. F. (2011). Revised recommendations on screening for chloroquine and hydroxychloroquine retinopathy. *Ophthalmology*.

[5] Maturi R. K., Yu M., Weleber R. G. (2004). Multifocal electroretinographic evaluation of long-term hydroxychloroquine users. *Archives of Ophthalmology*.

[6] Lai T. Y. Y., Chan W.-M., Li H., Lai R. Y. K., Lam D. S. C. (2005). Multifocal electroretinographic changes in patients receiving hydroxychloroquine therapy. *American Journal of Ophthalmology*.

[7] Tsang A. C., Pirshahid S. A., Virgili G., Gottlieb C. C., Hamilton J., Coupland S. G. (2015). Hydroxychloroquine and chloroquine retinopathy: a systematic review evaluating the multifocal electroretinogram as a screening test. *Ophthalmology*.

[8] Lyons J. S., Severns M. L. (2007). Detection of early hydroxychloroquine retinal toxicity enhanced by ring ratio analysis of multifocal electroretinography. *American Journal of Ophthalmology*.

[9] Yoon Y. H., Cho K. S., Hwang J. J., Lee S.-J., Choi J. A., Koh J.-Y. (2010). Induction of lysosomal dilatation, arrested autophagy, and cell death by chloroquine in cultured ARPE-19 cells. *Investigative Ophthalmology and Visual Science*.

[10] Bernstein H. N. (1983). Ophthalmologic considerations and testing in patients receiving long-term antimalarial therapy. *The American Journal of Medicine*.

[11] Rosenthal A. R., Kolb H., Bergsma D., Huxsoll D., Hopkins J. L. (1978). Chloroquine retinopathy in the rhesus monkey. *Investigative Ophthalmology and Visual Science*.

[12] Turgut B., Turkcuoglu P., Koca S. S., Aydemir O. (2009). Detection of the regression on hydroxychloroquine retinopathy in optical coherence tomography. *Clinical Rheumatology*.

[13] Browning D. J. (2013). Impact of the revised american academy of ophthalmology guidelines regarding hydroxychloroquine screening on actual practice. *American Journal of Ophthalmology*.

[14] Browning D. J., Lee C. (2014). Relative sensitivity and specificity of 10-2 visual fields, multifocal electroretinography, and spectral domain optical coherence tomography in detecting hydroxychloroquine and chloroquine retinopathy. *Clinical Ophthalmology*.

[15] Farrell D. F. (2012). Retinal toxicity to antimalarial drugs: chloroquine and hydroxychloroquine: a neurophysiologic study. *Clinical Ophthalmology*.

